# Analysis of the TGF-β1 of a Tibetan Plateau *Schizothoracine* Fish (*Gymnocypris dobula*) Revealed Enhanced Cytoprotection in Hypoxic Environments

**DOI:** 10.3390/genes16101176

**Published:** 2025-10-10

**Authors:** Ziyu Le, Xiaohui Wu, Yang Liu, Qianghua Xu, Congcong Wang

**Affiliations:** 1 College of Marine Living Resource Sciences and Management, Shanghai Ocean University, Shanghai 201306, China; 23210700105@m.fudan.edu.cn (Z.L.); wgxodn@164.com (X.W.); qhxu@shou.edu.cn (Q.X.); 2 School of Life Sciences, Fudan University, Shanghai 200438, China; 3 Marine Biomedical Science and Technology Innovation Platform of Lin-Gang Special Area, Ministry of Agriculture and Rural Affairs, Shanghai 201306, China; yliu@shou.edu.cn; 4 National Distant-Water Fisheries Engineering Research Center, Shanghai Ocean University, Shanghai 201306, China; 5 Key Laboratory of Sustainable Exploitation of Oceanic Fisheries Resources, Ministry of Education, Shanghai 201306, China

**Keywords:** TGF-β1, *Gymnocypris dobula*, Schizothoracinae, hypoxic adaptation

## Abstract

**Background:** The Tibetan Plateau, which is known for its high elevation and low oxygen levels, presents a challenging environment for its inhabitants. To adapt to these hypoxic conditions, species of *Schizothoracine*, a subfamily of *Cyprinidae*, have developed unique physiological mechanisms and functions. Transforming growth factor-β (TGF-β) is a multifunctional cytokine involved in the regulation of cell growth, differentiation, apoptosis, and the cellular immune response. However, its specific role in adaptation to hypoxia remains poorly understood. **Methods:** In this study, we aimed to characterize the TGF-β1 gene in *Gymnocypris dobula* (*Gd*) and *Schizothorax prenanti* (*Sp*) and to test whether TGF-β1 contributes to hypoxia adaptation in plateau *Schizothoracine* fish. The predicted protein for *Gd*-TGF-β1 contains several primary domains, including cwf21 (cdc5 protein 21), GYF (Glycine-Tyrosine-Phenylalanine), FN1 (Fibronectin 1), a conservative domain, and a signal peptide. **Results:** The results of tissue distribution revealed that the mRNA level of TGF-β1 in brain, heart, muscle, skin, gills, and spleen—which are key tissues involved in oxygen sensing, transport, and physiological adaptation to hypoxic environments—was significantly lower in *G. dobula* than that in *S. prenanti*. Western blotting analysis revealed that the expression of activated TGF-β1 in *G. dobula* was significantly higher than that in *S. prenanti*. To investigate whether TGF-β1 in *G. dobula* possesses hypoxic adaptive features, *Gd*-TGF-β1 and *Sp*-TGF-β1 were cloned into an expression vector and transfected into 293-T cells, which are widely used due to their ease of culture, high transfectability, and well-characterized properties. We found that the survival rate of cells transfected with *Gd*-TGF-β1 was significantly higher than that of cells transfected with *Sp*-TGF-β1 after hypoxia treatment. **Conclusions:** These findings suggest that *G. dobula* may promote hypoxic adaptation through the activation and increased expression of TGF-β1. Changes in TGF-β1 expression may play a role in the adaptation of *G. dobula* to hypoxic conditions.

## 1. Introduction

The Tibetan Plateau, situated in southwest China, boasts an average elevation exceeding 4000 m and is known as the Roof of the World. This region experiences a cold and arid climate with thin air that impacts the physiological functions of organisms substantially. Fish absorb dissolved oxygen from the water through their gills for respiration, which is essential for maintaining their basic physiological activities and survival [1]. Low dissolved oxygen levels can lead to fish stress, impaired growth, and even death [2]. The average dissolved oxygen content of lakes on the Tibetan Plateau is only 5.38 mg/L [3]. Consequently, fish inhabiting high altitudes have developed adaptive strategies enabling them to thrive and reproduce in such extreme environments.

Schizothoracinae are indigenous fish species found in the Qinghai–Tibet Plateau and belonging to the *Cyprinidae* family. Schizothoracinae is primarily categorized into 12 genera, and its morphology varies with altitude. As the plateau underwent uplift, Schizothoracinae evolved into three groups: primitive, intermediate, and advanced. *G. dobula*, a plateau Schizothoracinae, inhabits lakes and the surrounding waters of plateau regions such as the Peiku and Qucolong Lakes in Tibet. HIF-1α (Hypoxia inducible factor 1 alpha) may play a crucial role in mediating the evolutionary adaptation of Schizothoracinae in high-altitude environments through the nitric oxide synthase pathway [4]. In addition, Xu et al. (2016) observed that *G. dobula* adapts to high-altitude hypoxia through the upregulation of EPO (erythropoietin) expression and an increase in hemoglobin levels [5]. Notably, a key mutation in EPO, identified as H131S, enhances hypoxic adaptation by promoting apoptosis [6]. The aforementioned studies demonstrate that in the long-term evolution of plateau Schizothoracinae, *G. dobula* undergoes gene evolution and acquires new genes to adapt to extreme environments characterized by low pressure, perennial low temperature, and low oxygen content. These findings highlight the various mechanisms employed by organisms to adapt to extreme altitude conditions.

The TGF-β superfamily, a class of multi-effect cytokines, plays crucial regulatory roles in tissue repair, embryonic development, cell growth and differentiation, extracellular matrix, angiogenesis, apoptosis, and immune function [7]. It comprises TGF-β1, TGF-β2, TGF-β3, and TGF-β6, with TGF-β1 exhibiting the most extensive expression. TGF-β1 has been implicated in the transcriptional regulation of human fibroblasts, proliferation of the lung cancer cell line A549, cell growth and differentiation, and tissue damage repair [8,9,10], exerting a significant protective effect on cells. Furthermore, it has been demonstrated that TGF-β1 is activated by the SMAD (suppressor of mother against decapentaplegic) protein and other pathways when stimulated by extrinsic factors. TGF-β1 mediates signaling from the cell membrane to the nucleus under external stimulation by activating TGF-β-II and TGF-β-I receptors, which leads to SMAD protein phosphorylation [11]. Upon external stimulation, neuropeptide Y activation of the PI3K pathway via the Y1 receptor promotes TGF-β1 production in RAW264.7 cells, and ERK1/2 and RSK1 activate the antiapoptotic activity of TGF-β1 [12]. Additionally, hepatic stellate cells activate and elevate TGF-β1 expression in response to liver injury [13]. These studies illustrate that organisms can activate TGF-β1 and increase its expression to adapt to external stimulation.

Recently, numerous studies have revealed a close relationship between TGF-β1 and biological adaptation to hypoxia. Many in vivo experiments have shown that acute and chronic hypoxia induced by central nervous tissue ischemia, renal ischemia, liver cirrhosis, pulmonary fibrosis, fracture, trauma, and renal transplantation can upregulate TGF-β1 gene expression [14,15,16,17,18,19,20,21,22,23,24,25]. These findings suggest that increased expression of TGF-β1 can alleviate local hypoxia. Studies have shown that hypoxic treatment leads to increased transcriptional expression of TGF-β1 in *Oreochromis niloticus* [26]. In *Oncorhynchus mykiss*, treatment with bovine TGF-β1 resulted in an enhanced respiratory burst response [27]. Additionally, exposure to hypoxia for 6 h significantly raised TGF-β1 transcription levels in *O. mykiss* [28]. These findings indicate that TGF-β1 is crucial for the adaptation of fish to hypoxic conditions. However, the role of TGF-β1 in the hypoxic response and the molecular mechanisms involved in the response are relatively unexplored. Hypoxia adaptation involves multiple signaling pathways. In addition to the well-characterized HIF-1α pathway, TGF-β1 has been shown to participate in hypoxia-related processes such as fibrosis, apoptosis, and immune modulation through crosstalk with hypoxia-inducible factors. For example, HIF-1α can promote TGF-β1/Smad signaling under hypoxic conditions [29], while TGF-β1 itself has been reported to upregulate HIF-1α expression even under normoxia [30]. Nevertheless, its specific role in fish adaptation to long-term hypoxia remains poorly investigated.

In this study, cDNA sequences of the TGF-β1 gene of *G. dobula* and *S. prenanti* (another Schizothoracinae species inhabiting low altitudes) were determined. Subsequently, we analyzed the homology of the nucleotide and amino acid sequences. Moreover, we investigated the role of TGF-β1 of these fish in enhancing cell viability under hypoxic conditions. Our findings suggest that TGF-β1 may contribute to hypoxia adaptation in Schizothoracinae, providing a new perspective on their adaptive mechanisms.

## 2. Materials and Methods

### 2.1. Ethics Statement

This study involved fish samples collected directly from their natural wild habitat. No laboratory rearing or experimental interventions were performed. According to the National Standard of the People’s Republic of China (GB/T 35892-2018 [31], Guidelines on Ethical Review of Laboratory Animal Welfare), such work does not fall within the scope of “laboratory animal use” and therefore does not require formal approval by an institutional ethics committee. All procedures complied with the relevant national guidelines to minimize stress and suffering. Each specimen was ethically sacrificed by immersion in a eugenol solution (0.25 mL/L), in accordance with the Chinese guidelines for the ethical review of laboratory animal welfare (GB/T 35892-2018 [31]).

### 2.2. Sample Collection and RNA Isolation

Samples of the specialized *Schizothoracine* fish *G. dobula* were captured from Duoqing Lake, Tibet (28°03.371′ N, 89°17.831′ E; 4506 m above sea level). The habitat conditions included a temperature of 11.0 ± 0.2 °C, dissolved oxygen of 1.9 ± 0.3 mg/L, and a pH value of 8. *S. prenanti* (primitive *Schizothoracine* fish) was collected from Ya’an in Sichuan Province (at 950 m elevation; T 18.0 ± 0.2 °C; DO 9.0 ± 0.5 mg/L; pH 8). The fish were euthanized by immersion in an aqueous solution of eugenol (0.25 mL/L), following the laboratory animal guidelines for the ethical review of animal welfare in China (GB/T 35892-2018 [31]), and the tissues were quickly biopsied and placed in RNAlater (QIAGEN, Hilden, Germany) and stored at −80 °C until RNA extraction.

### 2.3. Cloning and Sequencing of TGF-β1 cDNA

We isolated total RNA from tissue samples of *G. dobula* and *S. prenanti* using TRIzol Reagent (Life Technologies, Carlsbad, CA, USA) following the manufacturer’s protocol and determined the concentration. Based on the sequence of the TGF-β1 gene of *G. dobula* and *S. prenanti* obtained from earlier transcriptome sequencing in our laboratory, we designed primers to obtain the complete open reading frames (ORFs) of *TGF-β1* using PCR (Table 1). PCR amplification began with a 5-min pre-denaturation step, followed by 30 thermal cycles consisting of 95 °C for 45 s (denaturation), 58 °C for 45 s (annealing), and 72 °C for 90 s (extension). A final elongation was performed at 72 °C for 10 min, after which the samples were held at 4 °C. The amplicons of expected length were ligated into the pMD18-T vector and subjected to sequencing.

### 2.4. Sequence Alignment

The nucleotide sequence of *Gd*-TGF-β1 was translated in silico (NCBI, http://www.ncbi.nlm.nih.gov/gorf/gorf.html, accessed on 8 March 2018). The nucleotide and putative amino acid sequence identities were verified using the BLAST program (http://blast.ncbi.nlm.nih.gov/Blast.cgi, accessed on 8 March 2018). The protein domains were marked using SMART (http://smart.embl-heidelberg.de/, accessed on 8 March 2018) and conserved domain database. Amino acid sequences were aligned using Clustal X 2.1 software. The amino acid sequences of *Gd*/*Sp*-TGF-β1 obtained by sequencing were compared with other fish (*Danio rerio*, *Cyprinus carpio*, *Carassius auratus*, *Onychostoma macrolepis*, *Sinocyclocheilus rhinocerous, Cirrhinus molitorella*, *Rhinichthys klamathensis goyatoka*, *Hemisalanx prognathous Regan*, *Chasmistes brevirostris*, *Takifugu rubripes*, *Oreochromis mossambicus*, *Oryzias melastigma*) to construct a phylogenetic tree by Mega 11.0 software. Mega 11.0 software was also used to calculate genetic distance. Statistical analyses were performed using R 4.2.3 software. Statistical significance was determined using one-way analysis of variance. * indicates *p* < 0.05, ** indicates *p* < 0.01.

### 2.5. Quantitative Real-Time PCR

We extracted high-quality RNA from six tissues (brain, heart, muscle, skin, gills, and spleen) of *G. dobula* and *S. prenanti*. From these samples, mRNA was isolated using magnetic oligo (dT) beads (Takara, Dalian, China). The purified mRNA was then denatured at 65 °C and served as the template for first-strand cDNA synthesis via reverse transcription. For the quantitative real-time PCR (qRT-PCR), specific primers were designed based on the TGF-β1 cDNA sequences of the two *Schizothoracine* species, with tubulin-1 and β-actin, both have been validated as relatively stable housekeeping genes under hypoxic or stress conditions in teleost fish and related aquatic organisms [32,33,34], serving as internal reference genes (Table 2). We quantified the TGF-β1 transcript levels on a CFX96™ Real-Time System (BIORAD, Hercules, CA, USA) using a SYBR^®^ Premix Ex Taq™ kit (Takara, Dalian, China). The thermal cycling protocol began with an initial denaturation at 95 °C for 30 s, followed by 40 cycles of denaturation at 95 °C for 5 s and a combined annealing/extension step at 58 °C for 45 s. A final melting curve analysis was performed to ensure PCR specificity. All reactions were conducted in triplicate, and the resulting data were analyzed using the normalized gene expression method (2^−ΔΔCT^).

### 2.6. Transient Expression of Gd-TGF-β1 and Sp-TGF-β1 in 293-T Cells

To construct the expression vectors, the coding sequences for *Gd*-TGF-β1 and *Sp*-TGF-β1 were released from the pMD18-T vector via digestion with *EcoRI* and *KpnI*. These fragments were then ligated into the pTOL2-bactin-2A-EGFP vector, which had been linearized with the same restriction enzymes. The resulting recombinant plasmids were purified using the OMEGA EndoFree Plasmid Midi Kit (Omega Bio-Tek, Shanghai, China) in preparation for cell transfection. For these experiments, Human Embryonic Kidney (HEK) 293-T cells were maintained in DMEM supplemented with 10% heat-inactivated fetal bovine serum and 1% penicillin/streptomycin. The cells were incubated for 48 h at 37 °C in a humidified atmosphere of 5% CO_2_. The HEK 293-T cells were inoculated into a six-well plate and transfected when the cell density reached 75–90%. The transfection system was as follows: 2 μg pTOL2-bactin-2A-EGFP, pTOL2-bactin-2A-EGFP-*Gd*-TGF-β1, and pTOL2-bactin-2A-EGFP-*Sp*-TGF-β1 plasmids and 6 μL XtremeGENE HP DNA Transfection Reagent (Roche, CH) were dissolved in 200 μL of DMEM. Six hours later, a fresh growth medium was added and cultured for 24 h.

### 2.7. Western Blotting

The head kidney is considered the primary hematopoietic organ and is responsible for the generation of blood cells and immune responses [35]. Therefore, Protein extraction was performed on head kidney of *S. prenanti* and *G. dobula*. In this study, the head kidneys of *S. prenanti* and *G. dobula* (three samples per species, 50–100 mg each, with three experimental replicates) were lysed with 600 μL of lysis buffer (990 μL RIPA + 10 μL PMSF). After 5 min, samples were centrifuged at 12,000 rpm at 4 °C for 15 min, and the supernatant was collected. A quarter volume of 4× SDS loading buffer was added, and samples were boiled for 15 min and stored at −20 °C. For protein expression detection, a 12% PAGE-SDS gel was used for electrophoresis, followed by transfer to a PVDF membrane (0.45 μm) at 0.3 A for 1 h. The membrane was blocked in 15 mL of 5% non-fat dry milk at room temperature for 2 h. After blocking, the TGF antibody (come from rabbit, Hangzhou HuaAn Biotechnology Co., Ltd., Hangzhou, China, Catalog: ET1701-97) was diluted 1:1000 in 5% non-fat dry milk and incubated at 4 °C, with three washes in PBST at 58 rpm for 6 min each. The secondary antibody was diluted 1:3000 in PBST and incubated at room temperature for 1 h, followed by three washes in PBST. ECL detection solution was applied to the membrane, and protein bands were observed using a protein imaging system.

### 2.8. Cell Viability Assay

The HEK 293-T cells were spread in a 96-well plate, and when the cell confluence was approximately 70–90%, the cells were transfected. The transfection system was as follows: 1 μg pTOL2-bactin-2A-EGFP, pTOL2-bactin-2A-EGFP-*Gd*-TGF-β1, and pTOL2-bactin-2A-EGFP-*Sp*-TGF-β1 plasmids and 3 μL transfection reagent were dissolved in 100 μL of DMEM, mixed well, placed on an ultraclean bench for 20 min, and then added into a 96-well plate according to the mixing reagent with a droplet size of 10 μL per well. The medium in the 96-well plate was replaced with a mixture containing 10 μL of CCK 8 reagent (Dojindo, Kumamoto-ken, Japan) and 90 μL of phenol red-free medium, incubated for 45 min at 37 °C in an incubator, and the fluorescence value was measured at 450 nm in each well using an enzyme marker. Cell viability was calculated using the following formula:

$$Cell\;viability=\frac{\left(fluorescence\;value\;from\;transfected\;cells-fluorescence\;value\;of\;fresh\;DMEM\right)}{\left(fluorescence\;value\;of\;non\text{-}transfected\;cells-fluorescence\;value\;of\;fresh\;DMEM\right)}\times 100\%$$

### 2.9. Statistical Method

All data were expressed as mean ± SD from three independent experiments. Statistical significance (*p* < 0.05) was assessed using one-way analysis of variance (ANOVA) followed by Tukey’s HSD tests, performed with SPSS Statistics 17.0 software (SPSS, Chicago, IL, USA).

## 3. Results

### 3.1. TGF-β1 Characteristics

**Figure 1:**
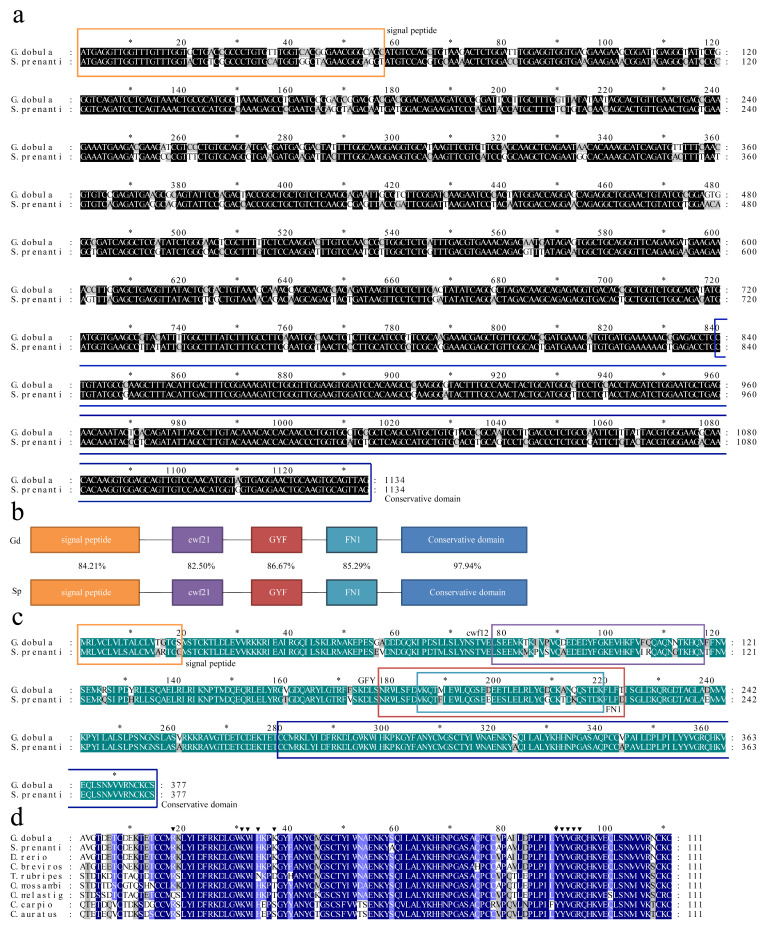
Characterization of *Gd*-TGF-β1 genes and phylogenetic analysis (**a**) Alignment of the ORF sequences of TGF-β1 with *G. dobula* and *S. prenanti*. Conserved residues are indicated in black, while variable regions are highlighted in gray. (**b**) Functional domain structures of TGF-β1 between *G. dobula* and *S. prenanti*. The amino acid positions are divided into several domains, including cwf21, GYF, FN1, conservative domain, signal peptide. (**c**) Alignment and comparison of deduced amino acid sequences of TGF-β1 from *G. dobula* and *S. prenanti*. Sequence identity is shown in green and the variable region is shown in gray. (**d**) Multiple amino acid sequence comparison of TGF-β1 in different species. Dark blue regions indicate amino acid residues that are 100% conserved in all species. Triangles indicate amino acid residues that are highly conserved in the binding region of TGF-β1 to its receptor. Asterisks indicate nine conserved cysteine residues that contribute to the “cysteine ring” in TGF-β1.

The ORFs of *S. prenanti*-TGF-β1 and *G. dobula*-TGF-β1 were 1134 bp, encoding 337 amino acids. The homology between *Gd*-TGF-β1 and *Sp*-TGF-β1 was 87.83% (Figure 1a). The predicted molecular weight of *Gd*-TGF-β1 protein was 43.25 kDa with an isoelectric point of 8.42, whereas that of *Sp*-TGF-β1 protein was 43.13 kDa with an isoelectric point of 8.63. The amino acid sequence homology of the TGF-β1 of the two fish was 89.66%, indicating a relative conservation of the amino acid sequence of the TGF-β1 of the plateau Schizothoracinae. The predicted *Gd*-TGF-β1 and *Sp*-TGF-β1 protein contains five typical structural domains: signal peptide (1–19), cwf21 (cdc5 protein 21, 78–117) [36], GYF (Glycine-Tyrosine-Phenylalanine domain, 178–223) [37], FN1 (Fibronectin type 1 domain, 185–219) [36], and conserved structural domains (280–337) (Figure 1b). Comparing these structural domains individually, the sequence similarity between *Gd*-TGF-β1 and *Sp*-TGF-β1 was 84.21%, 75.86%, 86.67%, 85.29%, and 97.93%, respectively (Figure 1c). In addition, we observed that nine invariant cysteine residues were present in the amino acids of *Gd*-TGF-β1 and *Sp*-TGF-β1 (Figure 1d).

### 3.2. Results of TGF-β1 Expression

**Figure 2:**
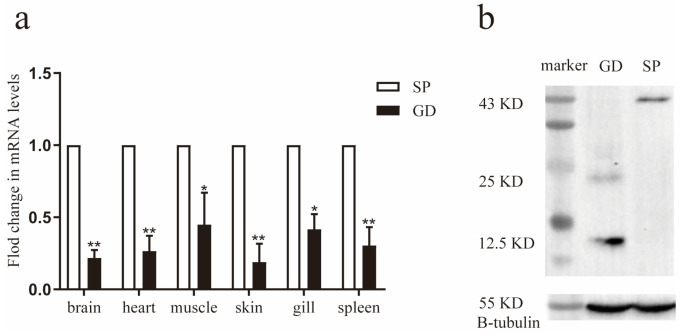
The protein and gene expressions of TGF-β1 between *S. prenanti* and *G. dobula* (**a**) Six tissues (brain, heart, muscle, skin, gill and spleen) from *G. dobula* (*Gd*) and *S. prenanti* (*Sp*) were investigated by qRT-PCR. Error bars indicated the mean ± SD (n = 3) in figures. * *p* < 0.05, ** *p* < 0.01. Statistical significance was determined by one-way ANOVA. The expression values of *S. prenanti* (*Sp*) were standardized to 1.0 and therefore no error bars are shown for *Sp*. (**b**) Western blotting was used to detect the protein levels of TGF-β1 in head kidney tissue sections from *G. dobula* and *S. prenanti*. 43 KD is TGF-β1 precursor and 25 KD is activated TGF-β1.

We investigated the transcript levels of TGF-β1 in various tissues of *G. dobula* and *S. prenanti* using relative fluorescence quantitative PCR. The findings revealed that TGF-β1 was expressed in the brain, heart, muscle, skin, gills, and spleen of both species. However, the mRNA level of TGF-β1 was notably lower in *G. dobula* than that in *S. prenanti* (Figure 2a).

Furthermore, we assessed the protein levels of TGF-β1 in the head kidney via Western blotting. We detected a 43 KD TGF-β1 precursor and a 25 KD activated TGF-β1. Interestingly, the expression of the activated TGF-β1 protein (25 KD) was significantly higher in *G. dobula* than that in *S. prenanti* (Figure 2b). These results suggest that during long-term acclimatization, TGF-β1 is activated to facilitate adaptation to hypoxic environments.

### 3.3. Hypoxic Adaptation of G. dobula-TGF-β1

**Figure 3:**
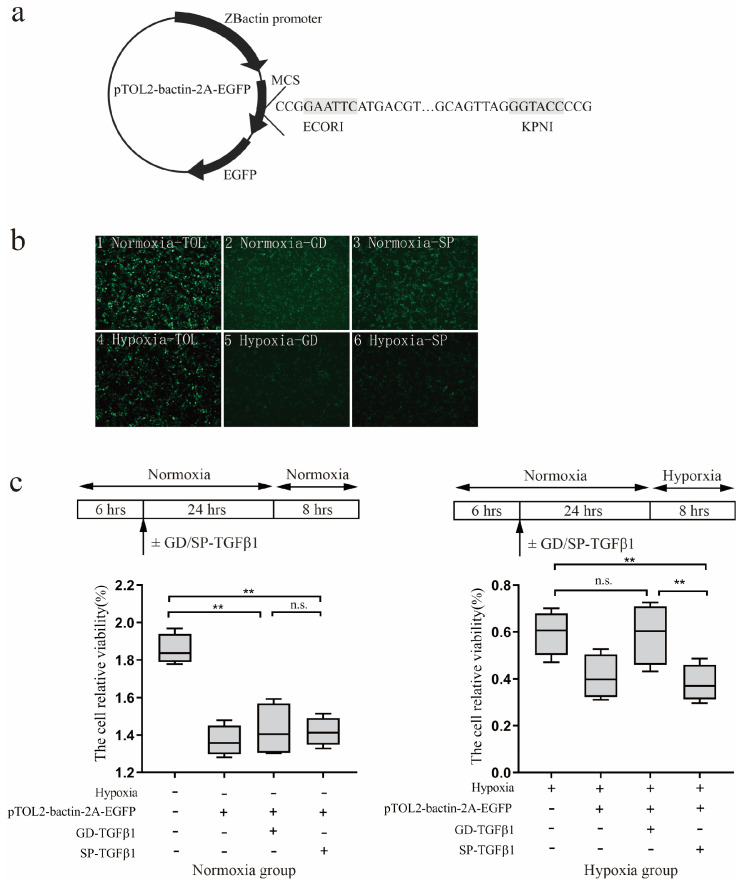
Viability analysis of 293T cells transfected with *schizothoracine* TGF-β1 under hypoxic challenge (**a**) The schematic drawing of recombinant TGF-β1 plasmid (**b**) Eukaryotic expression vectors of TGF-β1 between *G. dobula* and *S. prenanti* under normoxia and hypoxia. 1 mean eukaryotic expression vectors of Empty carrier of TOL2-bactin-2A-EGFP in normoxia; 2 mean eukaryotic expression vectors of TGF-β1of *G. dobula* in normoxia; 3 mean eukaryotic expression vectors of TGF-β1of *S. prenanti* in normoxia; 4 mean eukaryotic expression vectors of Empty carrier of TOL2-bactin-2A-EGFP under hypoxia; 5 mean eukaryotic expression vectors of TGF-β1 of *G. dobula* under hypoxia; 6 mean eukaryotic expression vectors of TGF-β1of *S. prenanti* under hypoxia. (**c**) The cell relative viability analysis of cells transfected with *schizothoracine* TGF-β1 under nomorxia and hypoxia. Error bars represent the mean ± SE (N = 8). * *p* < 0.05, ** *p* < 0.01. Statistical significance was determined by one-way ANOVA.

To explore the potential protective role of *G. dobula*-TGF-β1 against hypoxia, the TGF-β1 coding sequences of *G. dobula* and *S. prenanti* were cloned into expression vectors (Figure 3a) and subsequently transfected into HEK 293-T cells. The transfection efficiency was evaluated using fluorescence microscopy (Figure 3b). A substantial amount of green fluorescence was observed, indicating successful transfection of the eukaryotic expression vector.

In this study, we detected the cell survival using Alamar blue. As illustrated in Figure 3b, when exposed to hypoxia, cells expressing *G. dobula* TGF-β1 exhibited noticeably greater viability than those expressing *S. prenanti* TGF-β1. Consequently, *G. dobula* TGF-β1 appeared to effectively mitigate the decline in cell survival (Figure 3c). These results indicate distinct functional divergences between the two species’ TGF-β1 proteins, suggesting that the TGF-β1 gene in the high-altitude *Schizothoracine* species plays a protective role in low-oxygen environments.

## 4. Discussion

In this study, we compared the nucleotide sequences of TGF-β1 from *G. dobula* and *S. prenanti* and predicted their amino acid sequences. The amino acid similarity of TGF-β1 in plateau Schizothoracinae was 51–54% with *C. carpio*, *C. auratus*, and *Mylopharyngodon piceus* and 82.3% with *C. brevirostris* (Figure S1 and Table S1). This suggests that the amino acid sequence of TGF-β1 in plateau Schizothoracinae exhibits low interspecies variation and a high level of homology. Furthermore, we observed that the sequences of amino acids 280–377 were highly conserved at the C-terminus (Figure 2a). This conserved region has been identified as the main functional domain of the TGF-β1 protein, primarily regulating cell proliferation and differentiation, and has been associated with the development of various cardiovascular diseases [38]. TGF-β isoforms have nine invariant cysteine residues forming a “cysteine ring,” which has been suggested to be a common feature in fish [11]. In this study, we also identified these nine cysteine residues in the mature peptide of TGF-β1 from *G. dobula* and *S. prenanti*. Additionally, some research indicated that eight of these nine cysteine residues were present in TGF-β1 from grass carp, suggesting that this structural characteristic is conserved across all fish species [10]. This provides further evidence of the conserved nature of TGF-β1 in fish and predicts the functional similarity of TGF-β1 among fish species.

Currently, studies on the regulation of TGF-β1 under hypoxic stress in Schizothoracinae are very limited. To place our results into a broader physiological framework, we therefore compared them with findings from well-studied teleost models. Previous studies in species such as *C. carpio*, *C. auratus*, and *O. mykiss* have demonstrated that fish adapt to hypoxic environments by regulating the transcriptional expression of TGF-β1. Under hypoxic conditions, *C. carpio* exhibited higher expression levels of TGF-β1 in head kidney leukocytes, while *C. auratus* showed the highest transcript expression in the thymus, head kidney, and spleen [8,10]. In *O. mykiss*, higher TGF-β1 levels were noted in the spleen and thymus under hypoxic conditions, indicating that fish may respond to unfavorable environments by increasing TGF-β1 expression in the immune system [27]. Furthermore, under hypoxic conditions, zebrafish exhibited higher expression levels of *TGF-β1* in the eyes and gills but the levels were lower in the gut and muscle tissues [39]. These findings suggest that fish adapt to hypoxic environments by modulating the transcriptional expression of TGF-β1, with clear species- and tissue-specific variations in response. Consistent with this notion, our quantitative analysis showed that TGF-β1 mRNA levels were significantly lower in the brain, heart, muscle, skin, gills, and spleen of *G. dobula* compared with *S. prenanti*. However, Western blotting revealed that activated TGF-β1 protein expression was significantly higher in *G. dobula*, indicating that post-transcriptional activation mechanisms play a crucial role. Similar discrepancies between mRNA and protein abundance have been widely reported in other organisms, reflecting the influence of post-transcriptional and post-translational regulation [40,41]. TGF-β1 is secreted in an inactive (latent) form, forming a ring-shaped complex with its prodomain that shields it from recognition and maintains latency. The prodomain also contains an RGD sequence, recognized by αv integrins. Together, these two features—the protective “straitjacket” prodomain and the integrin-binding RGD motif—are key to TGF-β1 activation [42]. Under hypoxia, activation begins when latent TGF-β complexes are anchored to the extracellular matrix by latent TGF-binding proteins (LTBPs). Cell-surface αv integrins then bind the RGD sequence, and cytoskeletal forces induce conformational changes in the prodomain, releasing active TGF-β1. This active form binds to TGF-βRII dimers, recruits and phosphorylates TGF-βRI, which subsequently phosphorylates receptor SMADs (Smad2/3). The resulting complexes with Smad4 enter the nucleus, where they bind SMAD-responsive elements to regulate gene expression, particularly pathways linked to environmental adaptation such as the Akt/ARK5 system. For example, in human hepatocellular carcinoma (HepG2) cells, TGF-β activates Akt/ARK5 signaling to enhance tolerance to glucose deprivation under hypoxia [43]. Therefore, the higher activated protein levels despite lower mRNA expression suggest that *G. dobula* may adapt to hypoxia by enhancing post-transcriptional activation of TGF-β1, thereby promoting downstream gene activation.

Transient localized hypoxia, such as ischemia or trauma in tissues and organs of organisms, can contribute to TGF-β1 upregulation. In human hepatocellular carcinoma cells (HepG2), upregulation of TGF-β1 mRNA levels could enhanced cellular tolerance to hypoxic environments [42]. In fish, TGF-β1 also exhibited similar functions [10]. Additionally, transfection of the TGF-β1 gene from zebrafish embryos into 293-T cells, followed by exposure to 2.5% hypoxia for 24 h, showed a significantly higher survival rate in the transfected cells compared to controls [39]. These findings suggest that TGF-β1 acts as a hypoxia-responsive gene, providing protection to tissues and cells in adverse hypoxic environments. In this study, we observed that the survival rate of cells transfected with *G. dobula*-TGF-β1 under hypoxic conditions was significantly higher than that of cells transfected with *S. prenanti*-TGF-β1, suggesting that the altered expression of *G. dobula*-TGF-β1 may play a role in the acclimatization of plateau Schizothoracinae to hypoxic environments.

Although this study provides new insights into the potential role of TGF-β1 in hypoxia adaptation of Schizothoracinae, several aspects remain to be further explored. First, only two species (*G. dobula* and *S. prenanti*) were compared, which restricts the generalization of our conclusions to the entire Schizothoracinae subfamily. Future research should therefore include more species to assess the broader applicability of these findings. Second, we used HEK293T cells for functional validation because of their high transfection efficiency and stable performance, which makes them a widely used model system. However, these human-derived cells cannot fully represent the physiological environment of fish. Future studies should thus employ fish-derived cell lines or in vivo models to further verify the physiological relevance of our findings.

## 5. Conclusions

(1) The *G. dobula* TGF-β1 gene was characterized and shows high structural similarity to other fish species, indicating evolutionary conservation.

(2) Compared with *S. prenanti*, *G. dobula* shows lower TGF-β1 mRNA while exhibiting higher activated protein levels, indicating that protein activation plays a critical role in hypoxia adaptation.

(3) Functional assays demonstrated that *G. dobula* TGF-β1 enhances cell survival under hypoxic challenge, supporting its potential role in high-altitude adaptation.

(4) Although the specific adaptive functions and regulatory mechanisms of TGF-β1 under hypoxic conditions remain unclear, our findings offer new insights into the long-term adaptation of plateau-dwelling Schizothoracinae.

## Figures and Tables

**Table 1:** Gene primers for amplifying complete open reading frame.

Forward Primer	Reverse Primer	Sequences	Restriction Sites
*Gd*-TGF-β1-F		CCGGAATTCATGACGTTGGTTTGTTTG	ECORI
	*Gd*-TGF-β1-R	CGGGGTACCCTAACTGCACTTGCAGT	KPNI
*Sp*-TGF-β1-F		CCGGAATTCATGACGTTGGTTTGTTTG	ECORI
	*Sp*-TGF-β1-R	CGGGGTACCCTAACTGCACTTGCAGT	KPNI

**Table 2:** Primer sequences for qRT-PCR.

Name of Primers	Sequences (5′-3′)	Fragment Size (bp)
*Gd*-TGF-β1-F	AAGACTCTGGATTTGGAGGTGGT	171
*Gd*-TGF-β1-R	TTCGCTCAGTTCAACAGTGCTAT	
*Sp*-TGF-β1-F	CGAGTTACGGATTCGGATTA	112
*Sp*-TGF-β1-R	CTTGGAGACAAAGCGGGTGC	
Tubalin-1-F	AGTTTTGGTGGTGGCACCG	200
Tubalin-1-R	TTGTCCACCATGAAGGCACAGT	
β-actin-F	TGGCATCACACCTTCTACAACG	180
β-actin-R	AGAGGCATACAGGGACAGCACA	

## Data Availability

The data used to support the findings of this study are available from the corresponding author upon request.

## Supplementary Materials

The following supporting information can be downloaded at: https://www.mdpi.com/article/10.3390/genes16101176/s1, Figure S1: Evolutionary relationships of TGF-β1. The phylogenetic tree was constructed using the ML method; Table S1: The genetic distances between inferred TGF-β1 amino acid sequences in various species.

## References

[1] Davis J.C. (1975). Minimal dissolved oxygen requirements of aquatic life with emphasis on Canadian species: A review. J. Fish. Res. Board Can..

[2] Pollock M.S., Clarke L.M.J., Dubé M.G. (2007). The effects of hypoxia on fishes: From ecological relevance to physiological effects. Environ. Rev..

[3] Liu Z.Q., Pan B.Z., Han X., Li G., Wang T.Y. (2022). Water Environmental Characteristics and Water Quality Assessment of Lakes in Tibetan Plateau. Environ. Sci..

[4] Wang C.C., Wu X.H., Hu X.X., Jiang H.P., Chen L.B., Xu Q.H. (2020). Hypoxia-inducible factor 1α from a high-altitude fish enhances cytoprotection and elevates nitric oxide production in hypoxic environment. Fish Physiol. Biochem..

[5] Xu Q.H., Zhang C., Zhang D.S., Jiang H.P., Peng S.H., Liu Y., Zhao K., Wang C.C., Chen L.B. (2016). Analysis of the erythropoietin of a Tibetan Plateau *Schizothoracine* fish (*Gymnocypris dobula*) reveals enhanced cytoprotection function in hypoxic environments. BMC Evol. Biol..

[6] Wang C.C., Zhang Q., Liu Y., Xu Q.H. (2022). Characterization of EPO H131S as a key mutation site in the hypoxia-adaptive evolution of *Gymnocypris dobula*. Fish Physiol. Biochem..

[7] Gu J.Y., Gu X. (2002). Evolutionary analysis of functional divergence in TGF-β signaling pathway. Inf. Sci..

[8] Zhan Y., Jimmy K. (2000). Molecular isolation and characterisation of carp transforming growth factor beta 1 from activated leucocytes. Fish Shellfish Immunol..

[9] Gurneet K., Siqin H., Eric C., Tamara D.M., Jeffrey R., Chun P. (2003). Cloning of Transforming growth factor-beta 1(TGF-beta1) and its type I receptor from zebrafish ovary and role of TGF-beta l in ocyte maturation. Endocrinology.

[10] Yang M., Zhou H. (2008). Grass carp transforming growth factor-beta 1 (TGF beta1): Molecular cloning, tissue distribution and immunobiological activity in teleost peripheral blood lymphocytes. Mol. Immunol..

[11] Choi M.E. (2000). Mechanism of transforming growth factor-beta1 signaling. Kidney Int..

[12] Zhou J.R., Zheng X.U. (2008). Neuropeptide y promotes TGF-β1 production in raw264.7 cells by activating PI3K pathway via Y1 receptor. Neurosci. Bull..

[13] Williams E.J. (2001). Secretion and Activation of TGF-β1 by Hepatic Stellate Cells and Its Contribution to Liver Fibrosis. Ph.D. Thesis.

[14] Dhandapani K.M., Brann D.W. (2003). TGF-b1 neuroprotective factor in cerebral ischemia. Cell Biochem. Biophys..

[15] Docherty N.G., Perez-Barriocanal F., Balboa N.E. (2002). Transforming growth factor-beta1 (TGF-beta1): A potential recovery signal in the post-ischemic kidney. Ren. Fail..

[16] Papakonstantinou E., Aletras A.J., Roth M. (2003). Hypoxia modulates the effects of transforming growth factorbeta isoforms on matrix-formation by primary human lung fibroblasts. Cytokine.

[17] Lario S., Mendes D., Bescos M. (2003). Expression of transforming growth factor-beta1 and hypoxia-inducible factor-1 alpha in an experimental model of kidney transplantation. Transplantation.

[18] Warren S.M., Steinbrech D.S., Mehrara B.J. (2001). Hypoxia regulates osteoblast gene expression. Surg. Res..

[19] Jeong W.I., Do S.H., Yun H.S. (2004). Hypoxia potentiates transforming growth factor-beta expression of hepatocyte during the cirrhotic condition in rat liver. Liver Int. Off. J. Int. Assoc. Study Liver.

[20] Martinovic D., Villeneuve D.L., Kahl M.D., Blake L.S., Brodin J.D., Ankley G.T. (2009). Hypoxia alters gene expression in the gonads of zebrafish (*Danio rerio*). Aquat. Toxicol..

[21] Ju Z., Wells M.C., Heater S.J., Walter R.B. (2007). Multiple tissue gene expression analyses in Japanese medaka (*Oryzias latipes*) exposed to hypoxia. Comp. Biochem. Physiol. C Toxicol. Pharmacol..

[22] Zhang H., Hu Z., Li R., Wang Y., Zhou J., Xu H., Wang G., Qiu X., Wang X. (2022). Metabolomic Analysis of the *Takifugu obscurus* Gill under Acute Hypoxic Stress. Animals.

[23] Sand G.M., Diamond M.P. (2002). Hypoxia-indued irreversible up-regulation of type 1 collagen and transforming growth factor-beta l in human peritoneal fibroblasts. Fertil. Steril..

[24] Berger A.P., Kofler K., Bektic J., Rogatsch H., Steiner H., Bartsch G., Klocker H. (2003). Increased growth factor production in a human prostatic stromal cell culture model caused by hypoxia. Prostate.

[25] Maehr T., Costa M.M., Vecino J.L.G., Wadsworth S., Martin S.A.M., Wang T.H., Secombes C.J. (2013). Transforming growth factor-β1b: A second TGF-β1 paralogue in the rainbow trout (*Oncorhynchus mykiss*) that has a lower constitutive expression but is more responsive to immune stimulation. Fish Shellfish Immunol..

[26] Choi K., Lehmann D., Harms C., Law J.M. (2007). Acute hypoxia–reperfusion triggers immunocompromise in Nile tilapia. J. Aquat. Anim. Health.

[27] Jang S.I., Hardie L.J., Secombes C.J. (1994). Effects of transforming growth factor β1 on rainbow trout *Oncorhynchus mykiss* macrophage respiratory burst activity. Dev. Comp. Immunol..

[28] Aksakal E., Ekinci D. (2021). Effects of hypoxia and hyperoxia on growth parameters and transcription levels of growth, immune system and stress related genes in rainbow trout. Comp. Biochem. Physiol.—Part A Mol. Integr. Physiol..

[29] Wang H., Zhang Y., Cheng J. (2018). HIF-1α activates transforming growth factor-β1/Smad signaling in rat hepatic stellate cells under hypoxic conditions. Mol. Med. Rep..

[30] McMahon S., Charbonneau M., Grandmont S., Richard D.E., Dubois C.M. (2006). Transforming growth factor β1 induces hypoxia-inducible factor-1 stabilization through selective inhibition of PHD2 expression. J. Biol. Chem..

[31] (2018). Laboratory Animal—Guideline for Ethical Review of Animal Welfare.

[32] Chen X., Yu Y., Gao T., Liu Z., Chen S., Jia Y. (2025). Determination of Stable Reference Genes for Gene Expression Analysis in Black Rockfish (*Sebastes schlegelii*) Under Hypoxia Stress. Genes.

[33] Mohindra V., Tripathi R.K., Singh A., Singh R.K., Lal K.K. (2014). Identification of candidate reference genes for qRT-PCR under hypoxic stress in Clarias batrachus (Linnaeus, 1758). Int. Aquat. Res..

[34] Hu P., Liu M., Liu Y., Wang J., Lv Y. (2018). Identification of suitable reference genes for qRT-PCR analysis in yellow catfish *Pelteobagrus fulvidraco* under hypoxia stress. Aquac. Res..

[35] Kumar R., Joy K.P., Singh S.M. (2016). Morpho-histology of head kidney of female catfish *Heteropneustes fossilis*: Seasonal variations in melano-macrophage centers, melanin contents and effects of lipopolysaccharide and dexamethasone on melanins. Fish Physiol. Biochem..

[36] Wang J., Chitsaz F., Derbyshire M.K., Gonzales N.R., Gwadz M., Lu S., Marchler G.H., Song J.S., Thanki N., Yamashita R.A. (2023). The conserved domain database in 2023. Nucleic Acids Res..

[37] Michael K., Kathrin M., Christian F. (2005). GYF Domain Proteomics Reveals Interaction Sites in Known and Novel Target Proteins. Mol. Cell. Proteom..

[38] Santibañez J.F., Quintanilla M., Bernabeu C. (2011). TGF-β/TGF-β receptor system and its role in physiological and pathological conditions. Clin. Sci..

[39] Hou S.F. (2014). Expression Characteristics and Functional Analysis of the Hypoxia Response Gene TGF-β1 in Zebrafish. Master’s Thesis.

[40] Maier T., Güell M., Serrano L. (2009). Correlation of mRNA and Protein in Complex Biological Samples. FEBS Lett..

[41] Vogel C., Marcotte E.M. (2012). Insights into the Regulation of Protein Abundance from Proteomic and Transcriptomic Analyses. Nat. Rev. Genet..

[42] Shi M., Zhu J., Wang R. (2011). Latent TGF-β structure and activation. Nature.

[43] Suzuki A., Kusakai G., Shimojo Y., Chen J., Ogura T., Kobayashi M., Esumi H. (2005). Involvement of transforming growth factor-β1 signaling in hypoxia-induced tolerance to glucose starvation. J. Biol. Chem..

