# Supplementary material for: Analysis of the TGF-β1 of a Tibetan Plateau Schizothoracine Fish (Gymnocypris dobula) Revealed Enhanced Cytoprotection in Hypoxic Environments
Source: Genes (Basel). 2025 Oct 10;16(10):1176. doi: 10.3390/genes16101176 (PMC12562280; doi:10.3390/genes16101176)
Supplement: Supplementary file 1 [file genes-16-01176-s001.zip › genes-3904769-supplementary.pdf]

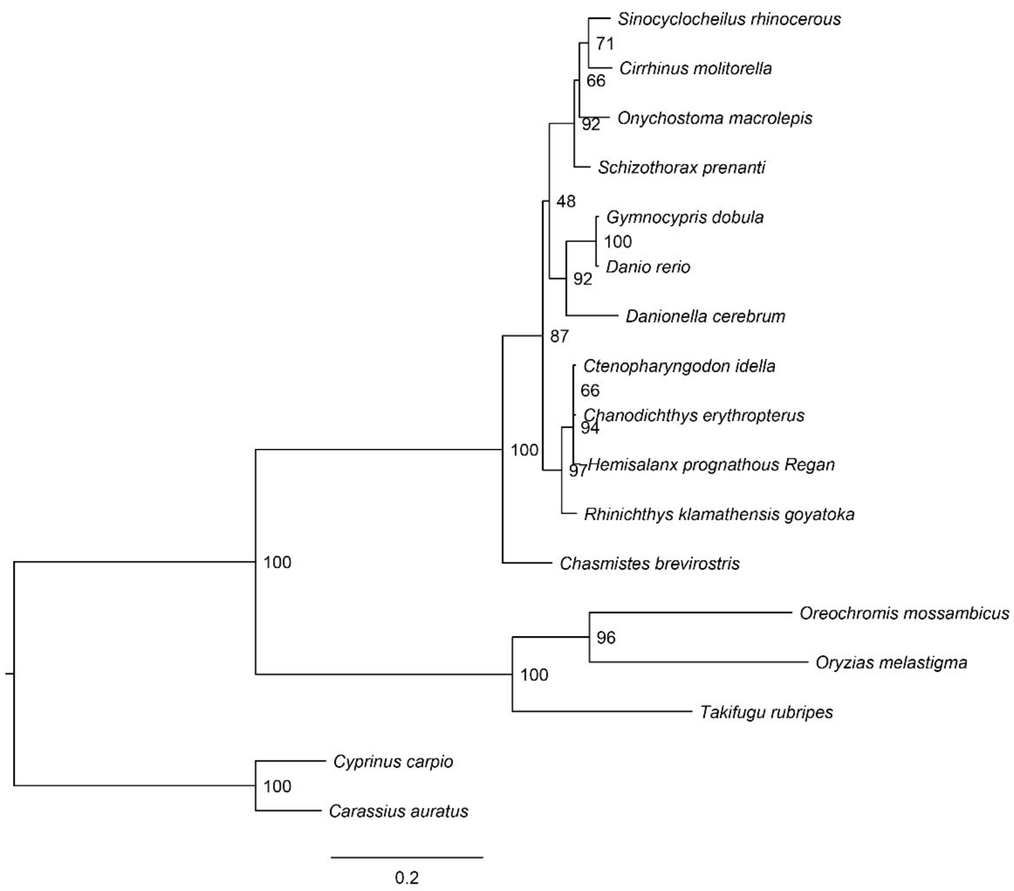

Figure S1. Evolutionary relationships of TGF-β1. The phylogenetic tree was constructed using the ML method.

Table S1. The genetic distances between inferred TGF-β1 amino acid sequences in various species

	Gymnocypris dobula	***Schizothorax prenanti***	***Danio rerio***	***Chasmistes brevirostris***	***Takifugu rubripes***	***Oreochromis mossambicus***	***Oryzias melastigma***	***Cyprinus carpio***	***Carassius auratus***
Gymnocypris dobula		0.01793	0.00359	0.02364	0.04603	0.04739	0.04975	0.04809	0.04880
Schizothorax prenanti	0.10920		0.01782	0.02027	0.04545	0.04623	0.04860	0.04791	0.04862
Danio rerio	0.00532	0.10920		0.02341	0.04615	0.04703	0.04984	0.04799	0.04872
Chasmistes brevirostris	0.18285	0.14535	0.18285		0.04469	0.04499	0.04762	0.04764	0.04894
Takifugu rubripes	0.58618	0.58136	0.59102	0.57034		0.03850	0.03869	0.04982	0.05019
Oreochromis mossambicus	0.61020	0.60524	0.61020	0.57866	0.46107		0.03738	0.05046	0.05007
Oryzias melastigma	0.61063	0.61063	0.61559	0.61105	0.44183	0.40147		0.04798	0.04774
Cyprinus carpio	0.63539	0.63539	0.64050	0.62794	0.65889	0.67176	0.65620		0.02226
Carassius auratus	0.64320	0.63809	0.64834	0.63569	0.67723	0.67976	0.65889	0.16802	
